# Replenishing the Aged Brains: Targeting Oligodendrocytes and Myelination?

**DOI:** 10.3389/fnagi.2021.760200

**Published:** 2021-11-25

**Authors:** Xi Zhang, Nanxin Huang, Lan Xiao, Fei Wang, Tao Li

**Affiliations:** ^1^Department of Histology and Embryology, Army Medical University (Third Military Medical University), Chongqing, China; ^2^Department of Ophthalmology, The General Hospital of Western Theater Command, Chengdu, China

**Keywords:** oligodendrocyte, OPC, myelinogenesis, aging, neurofunctional decline

## Abstract

Aging affects almost all the aspects of brain functions, but the mechanisms remain largely undefined. Increasing number of literatures have manifested the important role of glial cells in regulating the aging process. Oligodendroglial lineage cell is a major type of glia in central nervous system (CNS), composed of mature oligodendrocytes (OLs), and oligodendroglia precursor cells (OPCs). OLs produce myelin sheaths that insulate axons and provide metabolic support to meet the energy demand. OPCs maintain the population throughout lifetime with the abilities to proliferate and differentiate into OLs. Increasing evidence has shown that oligodendroglial cells display active dynamics in adult and aging CNS, which is extensively involved in age-related brain function decline in the elderly. In this review, we summarized present knowledge about dynamic changes of oligodendroglial lineage cells during normal aging and discussed their potential roles in age-related functional decline. Especially, focused on declined myelinogenesis during aging and underlying mechanisms. Clarifying those oligodendroglial changes and their effects on neurofunctional decline may provide new insights in understanding aging associated brain function declines.

## Introduction

Brain is sensitive to age with increasing neurofunction deficits including cognitive decline, motor and sensory abnormalities during aging (Sousounis et al., 2014; Damoiseaux, 2017; Jeromin and Bowser, 2017). Age-related impairments in cognition and memory lower the life quality of the elderly population and burden the society economically (Gazzaley et al., 2005). The neuron-loss hypothesis has been extensively tested; however, the neuron loss is obviously not the sole contributor for the severity of age-related functional decline (Morrison and Hof, 1997; Pakkenberg and Gundersen, 1997; Ihara et al., 2018). Noticeably, natural aging led to a reduction in white matter volume by as much as 28% (Liu et al., 2017). In addition, white matter abnormalities were identified and increased with age starting from middle age in humans (Kohama et al., 2012). White matter is mainly composed of bundled myelinated (87%) and unmyelinated axons and glia cells (Wang et al., 2008; Kohama et al., 2012). As the myelinating cells in the central nervous system (CNS), oligodendrocytes (OLs) are the most abundant glial cell type in white matter and also in some gray matter regions. For example, it was reported that OLs occupy about 75% of all glial cells in the neocortex of human brain (von Bartheld et al., 2016). More importantly, oligodendroglia lineage cells are undergoing dynamic changes during aging and that have been extensively reported recently (Stadelmann et al., 2019; Chapman and Hill, 2020; Sams, 2021).

Oligodendrocytes are exclusively derived from the differentiation of oligodendroglia precursor cells (OPCs), so does remyelination when demyelination occurs (Baumann and Pham-Dinh, 2001; Emery, 2010). OPCs distribute ubiquitously in the whole brain and have the capacities to proliferate and differentiate throughout lifetime. Each OL projects multiple processes to wrap the axons, forming the segmental, multiple-layered myelin sheaths that insulate axons. The denuded axon segment between two neighboring myelin sheaths is known as node of Ranvier, enriched with a number of ion channels. Action potential could travel along the axons quickly by jumping from one node of Ranvier to next one, so myelin could accelerate conduction velocity (Baumann and Pham-Dinh, 2001; Elbaz and Popko, 2019; Stadelmann et al., 2019). In addition, it is not hard to realize that through wrapping around the axon, myelin could protect the axon from damage. OLs may provide energy substance to axons via monocarboxylate transporter 1 (MCT1), and support axon survival (Fünfschilling et al., 2012; Lee et al., 2012; Morrison et al., 2013). Recent study suggested that myelin formation could regulate synaptic development in neonatal mouse brains (Wang et al., 2018).

Increasing evidence have demonstrated that myelinogenesis is continuously occurring in adult CNS and required for a number of neuro-functions in the adults, including memory function, motor coordination and motor skill learning (Young et al., 2013; McKenzie et al., 2014; Pan et al., 2020; Steadman et al., 2020; Wang et al., 2020; Chen L. et al., 2021). Because of the extremely high density of myelin sheaths in brains, dissecting the changes of oligodendroglial lineage cells and associated myelin during aging necessitates approaches to distinguish newly-formed myelin and pre-existing myelin. Recent advances by using cell-lineage labeling mouse lines and two-photon confocal imaging, allowed scientists to track the fate of oligodendroglial lineage cells and associated myelin during aging (Young et al., 2013; Baxi et al., 2017; Tripathi et al., 2017; Hill et al., 2018; Wang et al., 2020). This review aims to summarize recent evidence of dynamic changes of oligodendroglia lineage cells during aging and potential mechanisms of aging-related myelinogenesis decline. To that purpose, we first cover the development of oligodendroglia lineage cells and dynamic changes during aging. Next, we dissected the underlying mechanisms of inhibited myelination during aging. The pro-OPCs differentiation methods were also discussed as a potential therapy to improve age-related functional deficits.

## Oligodendrocyte Myelination in Adult Central Nervous System

Oligodendroglia precursor cells distribute into the whole brain and have the capacities to proliferate and differentiate throughout lifetime. Upon differentiation or apoptosis in either physiological or pathological conditions, the neighboring OPCs could proliferate rapidly and maintain the stable OPC density (Chang et al., 2000; Hughes et al., 2013; Sun et al., 2018; Bottes and Jessberger, 2021). Myelination is initiated after birth in rodents and reaches to a peak from 2 weeks to a month postnatally. The process is orchestrated by a large amount of intrinsic and extrinsic factors (Baumann and Pham-Dinh, 2001; Young et al., 2013; Elbaz and Popko, 2019). Notably, the time and extent of myelin formation is variable in different brain regions, which may be relative to the development of neuronal functions. For example, the lateral olfactory tract is myelinated the earliest in mouse brains, starting from postnatal day 4, while the axons in optic nerve start to be myelinated about 8 days postnatally. The superficial layer of cortex is the last region to be myelinated, where new myelin sheaths are continuously added into adulthood (Purger et al., 2016; Hill et al., 2018). Though it was proposed that programmed cell death of a subset of pre-myelinating OLs and excess myelin sheaths elimination by microglia are involved (Sun et al., 2018; Hughes and Appel, 2020), the exact mechanisms that regulate the temporal and spatial process are still unknown yet.

More and more evidence has demonstrated the generation of new OLs in adult and aging brains. EdU or BrdU incorporation assay showed that there were a number of EdU or BrdU positive mature OLs in the adult brains (Lasiene et al., 2009; Young et al., 2013; Gibson et al., 2014). Advances in cell-lineage labeling mouse line and two-photon confocal imaging contribute to observing new myelin generation in adults directly. Tau-mGFP is a transgenic reporter mouse line to label newly-formed myelin. After crossed with an OPC-specific Cre mouse line and tamoxifen induction, the mGFP is only highly expressed in OLs and associated myelin sheaths (Young et al., 2013; McKenzie et al., 2014; Wang et al., 2018). Numerous mGFP positive new myelin sheaths were observed in the motor cortex, corpus callosum, sensory cortex, and hippocampus of 10-months old brains in NG2-CreErt; Tau-mGFP mice after induction at the age of 7 months (Chen L. et al., 2021). New OLs and associated myelin were also observed in superficial cortex of adult and aged brains (Hill et al., 2018; Hughes and Orthmann-Murphy, 2018).

Several studies have demonstrated that active myelinogenesis in adults plays an important role in neurological function, including motor coordination, motor skill learning and memory function, as a concrete form of neuro-function plasticity (McKenzie et al., 2014; Xiao et al., 2016; Pan et al., 2020; Steadman et al., 2020; Wang et al., 2020; Chen L. et al., 2021). Inhibition of new myelin generation directly disrupts neuro-functions in adults. Transcriptional factors Olig2 and myelin regulating factor (Myrf) are known as positive factors to promote OPCs differentiation (Emery et al., 2009; Mei et al., 2013; Yu et al., 2013; Elbaz and Popko, 2019). Newly-formed myelin was significantly decreased in Olig2 or Myrf conditionally knockout mice (McKenzie et al., 2014; Xiao et al., 2016; Pan et al., 2020; Steadman et al., 2020; Wang et al., 2020; Chen L. et al., 2021). Importantly, neuro-function deficits were detected by different types of behavioral tests. For instance, the Olig2 knockout mice had more foot slips in a modified beam-walking test and less crossings in the rehearsal phase of water maze test (Wang et al., 2020; Chen L. et al., 2021). Water maze test and conditional contextual fear test indicated that adult Myrf knockout mice showed deficits in spatial memory consolidation and recalling of remote fear memory (Pan et al., 2020; Steadman et al., 2020). Conditionally deleting Myrf could impair motor skill learning function in adults (McKenzie et al., 2014; Xiao et al., 2016). In addition, recent studies revealed there was a new oligodendrogenic niche in the adult mouse median eminence, where the OLs differentiation was crucial for perineuronal net remodeling, which is also Myrf dependent (Zilkha-Falb et al., 2020; Kohnke et al., 2021). We speculated that losing the ability to accelerate action potential conduction along corresponding neuronal circuit may play a crucial role, and the underlying mechanisms of myelination in neuro-function plasticity needs further exploration.

## Age-Related Changes in White Matter and Oligodendroglial Cells

It is widely recognized that white matter alteration in rodents, monkeys and humans was greatly relevant to age-related neuro-functional decline. Critically, myelin pathology even emerges before neuronal change in normal aging animals (Pini et al., 2016; Hase et al., 2018; Nasrabady et al., 2018). Presently, there are large amount of imaging studies in both human and non-human primates showing white matter loss during aging (Tang et al., 1997; Kohama et al., 2012; Liu et al., 2017). Increasing histological studies further revealed the ultra-structural changes of myelin, while at the molecular level, the evidence about oligodendroglial change during aging is still limited.

### Age-Related White Matter Changes

Advances in neuroimaging contributes to explore macroscopical and microstructural changes in white matter (Ding et al., 2021; Kolb et al., 2021). Taking advantage of MRI imaging and diffusion tensor imaging (DTI), a variety of age-related white matter changes, including reduced white matter volume, white matter lesions, disrupted white matter integrity, and subsequent cortical disconnection have all been observed in normal aging brains (Caligiuri et al., 2015). It is reported that in humans, white matter volume gradually increases in the first 40 years of life, peaks at around 50 years of age, and then decreases rapidly from 60 years of age onward (Bennett and Madden, 2014; Liu et al., 2016). Even with healthy aging, white matter lesions (also known as leukoaraiosis) are evident as hyperintensities in white matter, which increase with age. The location of white matter lesions in different brain regions is in accordance with distinct types of functional decline. For example, white matter lesions in the frontal lobe, which is believed to be especially vulnerable to age-related white matter changes, are responsible for cognitive impairments. Subcortical white matter lesions are mainly correlated with depression in the elderly whereas periventricular white matter lesions are mainly related to cognitive decline (Bartrés-Faz et al., 2001; Barrick et al., 2010; Bennett and Madden, 2014). Moreover, the severity of white matter hyperintensities is correlated with the cognitive decline extent (Barrick et al., 2010; Cox et al., 2016; Bells et al., 2019). Similarly, degradation of white matter integrity and subsequent cortical disconnection revealed by DTI studies were reported in aging brain and are significantly associated with reduced cognitive function, including memory, executive function and general cognition (Bennett and Madden, 2014; Coelho et al., 2021).

### Age-Related Changes of Oligodendrocytes and Associated Myelin

As imaging results normally show a gross alteration of white matter, more and more histological studies are carried out to further confirm the underlying change of OLs and associated myelin sheaths. Both longitudinal live imaging and immunostaining give direct evidence that OL density, myelin segment length and myelin density undergo a steady increase but followed by a gradually obvious decrease (Young et al., 2013; Tripathi et al., 2017; Hill et al., 2018). A recent work in our lab also showed that in layers I–III of the motor cortex, myelin basic protein (MBP) intensity and OL number were significantly increased from 4 months to 13 months, though the pre-existing myelin was decreased by 10%, detected by a transgenic mouse line (PLP-CreErt; mT/mG). But the MBP intensity and OL number decreased steeply at 18 months, while OPCs number was unaltered (Wang et al., 2020). On the other hand, the number of myelin internodes maintained by individual cortical OLs is stable for at least 8 months but declines 12% in the following year (Tripathi et al., 2017). The average length of internodes also decreases with aging (Mukoyama, 1973; Lasiene et al., 2009; Hill et al., 2018).

More importantly, besides the quantity alteration, the structure of myelin also appears abnormal with aging. In aged rats, there is increased splitting of the myelin sheath, myelin balloon formation, and separation from the axon (Sugiyama et al., 2002; Attia et al., 2019). Aged CNS exhibit paranodal pilling that result in reorganization of the cluster of ion channels at the nodes of Ranvier, which would be detrimental to action potential conduction (Hinman et al., 2006; Shepherd et al., 2012). Meanwhile, ultra-structure of myelin also changes during aging. EM studies showed declined myelin thickness, myelin density and myelin fraction in aged marmosets (Phillips et al., 2019). In cingulate bundle and corpus callosum of rhesus monkeys, it was demonstrated that myelin sheath exhibited an increasing frequency of degenerative changes such as dense sheaths, myelin balloons and redundant sheaths during normal aging. Critically, the percentage of degenerative myelin was negatively associated with cognitive performance (Bowley et al., 2010). Although mature OLs and myelin sheath they formed are supposed to be stable, it is unavoidable that myelin debris will present in aging mice (up to 24 months), as electron microscopy images of white matter showed multilamellar myelin fragments in the extracellular space or inside of cells, immunohistochemistry further confirmed those myelin debris located in microglial cells (Safaiyan et al., 2016; Cantuti-Castelvetri and Fitzner, 2018). In live aged mice (910-day-old), formation of myelin spheroids, myelin debris and myelin loss were longitudinally recorded. It turned out that myelin spheroids occurred slowly over weeks, and once formed, some remained for at least a month (Hill et al., 2018).

**FIGURE 1:**
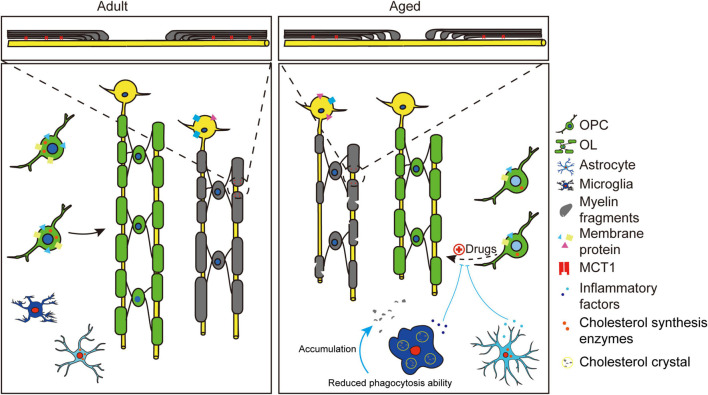
Schematic showing changes of OPCs and OLs during aging. Compared to adults, aged brains showed myelin degeneration and inhibited myelinogenesis. The pre-existing myelin (gray) showed quantitative and qualitative changes during aging. Quantitative alterations include decreased OLs number and internodes, thinner myelin sheaths and shortened internodes. Qualitative changes include myelin debris formation, microstructural changes like paranodal pilling (zoomed area) and altered membrane protein expression (decreased MCT1, decreased GLAST1, and increased NMDA). Inhibited myelinogenesis (green) may be due to the age-related intracellular and extracellular changes of OPCs. Intracellular changes include varied epigenetic regulation (light blue nucleus), decreased cholesterol synthesis enzymes and decreased receptor expression (GPR17, APJ). Extracellular changes include stiffness of extracellular matrix (ECM) and age-related activation of astrocytes and microglia cells. Proinflammatory factors (cytokines, chemokines) released by activated glial cells will inhibit differentiation of adult OPCs. Activated microglia cells are associated with cholesterol crystal formation (see yellow circles) and reduced phagocytic ability, resulting in myelin debris accumulation. Activated astrocytes show decreased cholesterol synthesis enzymes. Note that the inhibited differentiation of OPCs in aged brain could be rejuvenated by drugs like clemastine.

The above morphological changes all lead to a fact that at least some of the mature OLs in the aging brain is experiencing degeneration. There should be extensive alteration in those OLs, such as their functional protein expression, their metabolism state and their interaction with other glial cells and neurons (Sugiyama et al., 2002; Wang et al., 2014; Tse and Herrup, 2017). However, related evidence is limited. In agree with our perspective, a recent work using transgenic mice revealed that spinal cord myelin MCT1 protein expression had declined by 35% by the age of P360, when mice are considered middle age, corresponding to about 42-year-old in humans (Philips et al., 2021). It is accepted that in later stage of life, the ability of OLs to supply energy to axon will drop intensely, which may participate in broad neuronal functional deficits. As is reported that age-related decreases in lactic acid emerges in the hippocampus in the senescence-accelerated mouse, which might be linked with cognitive impairments (Wang et al., 2014). Meanwhile, the increase in oligodendroglial NMDA receptors and decrease in glial glutamate uptake transporter GLAST1 is found in aging white matter, which is believed to be detrimental to myelin and OLs (Rivera et al., 2016). In addition, the expression of several myelin proteins was shown to change with aging. For example, the absence of the 21.5-kDa isoform of MBP in aged mice or age-related dysregulation of cyclic nucleotide phosphodiesterase (Sugiyama et al., 2002; Sloane et al., 2003; Hinman et al., 2008). The two proteins are vital elements for myelin maintenance. It should also be noted that myelin is composed of ∼70% lipid and ∼30% protein. The decrease in lipid component (especially cholesterol) rather than protein content during aging was believed to be account for the loss of myelin in old monkeys (Baumann and Pham-Dinh, 2001; Sloane et al., 2003; Summarized in Figure 1).

Changes in OLs and associated myelin with aging will undoubtedly affect the neuronal transmission efficiency, the energy supply of axons and the vulnerability of axons. Meanwhile, myelin dysfunction during aging will impact on microglial functions and subsequently the micro-environment, which has been recently proved to be an upstream AD risk factor (Depp et al., 2021). Unfortunately, there was no effective strategy to inhibit or delay age-related myelin loss or degeneration up to now.

### Age-Related Changes of Oligodendroglia Precursor Cells

Increasing studies reveal that myelinogenesis decreases with normative aging. In the NG2-CreErt; Tau-mGFP mouse line, which could label newly formed myelin, there were abundant mGFP positive new OLs and myelin sheath in the cortex, corpus callosum and hippocampus of 6 or 8-months old brains. Correspondingly, new OLs and associated myelin was steeply decreased in the 18- or 22-months old brains (Wang et al., 2020). Considering functional importance of myelinogenesis in adults, it was accepted that decreased myelinogenesis played important role in age-related memory function deficit. For instance, the above mentioned 13-months old mice showed spatial learning and memory function decline. Inspiringly, genetical and pharmaceutical interventions are proved to be helpful. Muscarinic receptor 1 (M1R) was a negative regulator for OPCs differentiation, and clemastine was proved to promote myelin development and remyelination through M1R (Mei et al., 2016; Wang et al., 2018). Specially deleting M1R in aging OPCs or clemastine treatment could increase mGFP positive OLs and associated myelin in cortex, corpus callosum and hippocampus in the aged brains. Amazingly, aged M1R knockout and clemastine treated mice showed improved spatial memory function. It seemed that memory function recovery was associated with reversed synaptic loss in hippocampus (Wang et al., 2020). These indicated that promoting myelinogenesis may be a potential strategy to recover age-related functional decline.

It is generally acknowledged that decreased myelinogenesis distributes to inhibited OPCs differentiation or decreased progenitor cells. Recent evidence showed that the total number of OPCs remained stable in the aged brains. For instance, the number of OPCs in 10-year-old human corpus callosum is about 2∼3 × 10^8^ and it remains stable even up to 90 years (Yeung et al., 2014). Consistent with this, the number of OPCs didn’t have significant change in 18-month-old mouse cortex, comparing to the 4-month-old or 13-month-old mice (Wang et al., 2020). EdU or BrdU incorporation assays showed that NG2 or PDGFαR positive signal was co-expressed with EdU or BrdU during aging, suggesting that OPCs have reserved the ability to proliferate (Lasiene et al., 2009; Young et al., 2013). However, gene expression profile analysis found down-regulation of cell proliferation associated genes in OPCs during aging, accompanied with increased cell cycle time. Results from transgenic reporter mice showed proliferative OPCs in G2/M phase were obviously decreased in the aged brains (Spitzer et al., 2019). Though the proliferation ability of OPCs declined slightly with aging, total number of OPCs were stable. In a word, declined myelinogenesis is not likely caused by decreased OPCs.

Thus, it is widely accepted that decreased myelinogenesis during aging is attributed to declined differentiation capacity of the OPCs (Rivera et al., 2016; Hill et al., 2018; Wang et al., 2020). For instance, OPCs from the aged brains (18 months) differentiate slowly and have slower reaction to pro-differentiation compounds, compared to adult OPCs (2–3 months). Sequencing studies showed that aged OPCs have reduced OPC-specific gene expression and more markers of aging, including but not limited to mitochondrial dysfunction, unfolded protein response (UPR) and autophagy (Neumann et al., 2019a). Evidence from *in vitro* experiments also suggested inhibited differentiation of old OPCs (Neumann et al., 2019a). Moreover, the expression of several receptors which play essential roles in OPC differentiation and myelination are shown to change remarkably during aging, indicating the inhibited maturation ability of adult OPCs (Young et al., 2013; Spitzer et al., 2019). This will be further discussed later.

When demyelination occurred, remyelination contributed to myelin and neuro-function recovery. It was accepted that adult OPCs could migrate from adjacent or subventricular zone, proliferate and differentiate into mature OLs to form myelin (Smith et al., 1981; Deshmukh et al., 2013). Though surviving OLs were found to have the ability for myelin regeneration, it was demonstrated that newly-differentiated OPCs exhibited a much greater capacity for myelin regeneration after demyelination in zebrafish (Neely et al., 2020). In age -related neurodegenerative disease mouse model, including Alzheimer disease (AD) and Huntington’s disease, new OLs and associated myelin were remarkably increased in early stages likely to compensate myelin loss in lesions (Jin et al., 2015; Chen J. F. et al., 2021). Intriguingly, newly-formed myelin in AD mice was proven to increase progressively and contributed to functional recovery (Chen J. F. et al., 2021), suggesting that newly-formed myelin was quite stable in neurodegenerative diseases. However, myelin repair is usually not efficient in the site of injury. Especially, increasing evidence showed declined remyelination ability during aging (Sim et al., 2002; Goldschmidt et al., 2009). Multiple sclerosis (MS) is known as a neuroinflammatory and demyelination disease that is characterized by auto-immune mediated demyelination in the CNS, accompanied with secondary axon injury and neuro-function deficits (Franklin and Ffrench-Constant, 2008; Reich et al., 2018). OPCs are present within and around the demyelination lesions in the aged MS model mice, but fail to differentiate and form new myelin, suggesting that in those pathological context, age-related differentiation arrest of OPCs may also be an important cause of remyelination failure (Gilson and Blakemore, 1993; Sim et al., 2002; Goldschmidt et al., 2009; Ruckh et al., 2012). Interestingly, Metformin or LY294002 treatment, or environmental modulation by replacing young macrophages could recover the capacity of OPCs differentiation and remyelination in the aged mice (Ruckh et al., 2012; Neumann et al., 2019a; Rivera et al., 2021).

## Mechanisms of Inhibited Myelinogenesis During Aging

It is reported that myelin showed remarkable homeostatic resilience in adult mice. Recent works also pointed out that new myelin generation is highly active in adult mice, reflecting the strong differentiation ability of OPCs. Why the myelinogenesis ability dropped during aging? Here we summarized possible intrinsic and extrinsic mechanisms.

### Intrinsic Factors

#### Epigenetic Regulation

Increasing evidence suggested that the epigenetic regulators, including histone modifications and DNA methylation, played important roles in myelin development (Shen et al., 2005; Egawa et al., 2019). Histone deacetylases (HDACs) could remove acetyl groups from histone tails to regulate myelination associated genes expression (Liu et al., 2009; Conway et al., 2012). It was demonstrated that HDAC recruitment was inefficient in old brains in a cuprizone induced demyelination mouse model, compared to the adults, resulting in remyelination failure. Age-dependent HDAC1 recruitment to repressive complexes Hes5 and Sox2 promoters may be the main cause (Shen et al., 2008). Besides, DNA methylation analysis showed that OPCs from 16-months old brains were characterized by global hypomethylation and declined DNA methyltransferases activity, compared to adults (Zhou et al., 2019). These suggested decreased myelinogenesis or failure of remyelination was relative to changes of epigenetic regulation during aging.

#### Age-Related Protein Change in Oligodendroglia Precursor Cells

A recent study using proteomic analysis of OPCs isolated from the brains of neonatal, young and aged rat revealed that the amount of proteins associated with oxidative phosphorylation, inflammatory responses and actin cytoskeletal organization increased with age, whereas cholesterol-biosynthesis, transcription factors and cell cycle proteins decreased (de la Fuente et al., 2020). Besides, the receptors or ion channel alterations in OPCs in aged brains will directly affect the function of OPCs and their differentiation ability. For instance, genetic-fate mapping showed gradual decrease of GPR17 expression in OPCs in aging cerebrum. Moreover, in this study, GPR17 was believed to be a major factor affected during OL degeneration in the aging brain (Rivera et al., 2021). Apelin receptor (APJ) is a newly identified G-protein-coupled receptor, which could regulate OPCs differentiation through Myrf signal. The expression of APJ was decreased during aging, and level of apelin, the ligand of APJ, was also reduced significantly in the plasma of aged mice. More importantly, APJ activation could promote remyelination in aged mice (Ito et al., 2021). NMDAR-mediated signal play essential roles in myelination. Consistent with declined myelinogenesis, electrophysiological recordings showed remarkable decrease in NMDAR density in OPCs from 300-day old mice, compared to OPCs derived from adults or neonates (Spitzer et al., 2019). Although recent genomic analysis provided plenty of altered molecules in adult OPCs, the associated functional changes during natural aging should be further explored.

### Extrinsic Factors

#### Extracellular Signals

The elements and characters of microenvironment where OPCs lived changed with aging and the environmental changes may played an important role in age-related declined myelinogenesis. Firstly, the characters of microenvironment could regulate OPCs function. Atomic force microscopy showed that the prefrontal cortex progressively stiffened with aging. Seeding the adult OPCs in the aged decellularized brain extracellular matrix led to declined capacity of proliferate and differentiate, and the aged OPCs seeding in the adult brains could partially differentiate into mature OLs, suggesting that character of aged ECM impaired the OPCs function. Piezo1 is known as mechanosensitive ion channel, which could regulate cell density and stem cell activity. Knockdown of Piezo1 could recover the impaired OPCs differentiation in the aged brains (Neumann et al., 2019b; Segel et al., 2019). Secondly, molecules in extracellular matrix, such as hyaluronan and chondroitin sulfate proteoglycans, were negative regulators for effective remyelination in MS patients or EAE brains (Keough et al., 2016; Stephenson et al., 2019). These elements in ECM accumulated with aging, which may play an important role in the declined capacity of differentiation (Richard et al., 2018; Macke et al., 2020). It should be noted that neural stem cells in the subventricular zone (SVZ) also participate in myelin repair (Menn et al., 2006), while the output ability of those stem cells is similarly affected with the aging of SVZ niche (Luo et al., 2006; Bouab et al., 2011).

#### Age-Related Changes in Microglia and Astrocytes

Other glial cells, astrocytes and microglia, were known as regulators in myelination (Stadelmann et al., 2019). Age-related changes in astrocytes and microglia may play crucial roles in declined myelination/remyelination in the aged.

In the aging brain and in pathological conditions, microglia played an important role in clearing myelin pieces (Cignarella et al., 2020). In addition, microglia-derived transglutaminase-2 signals to GPR56 on OPCs could promote remyelination in murine models of demyelination (Giera et al., 2018). A recent study found that sterol synthesis in microglia/macrophages could resolve inflammation, which is essential for myelin repair (Berghoff et al., 2021). In aged mice, total number and density of microglia increased in various regions of the brain (Poliani et al., 2015). Myelin fragmentation increased with age and led to the formation of insoluble inclusions in microglia (Streit et al., 2004; Safaiyan et al., 2016; Rawji et al., 2018), accumulation of myelin debris in aged phagocytes led to cholesterol clearance deficits (Cantuti-Castelvetri and Fitzner, 2018). Moreover, those lipid droplet-accumulating microglia in the aging brain will produce high levels of reactive oxygen species, and secrete pro-inflammatory cytokines (Marschallinger et al., 2019). Single cell sequencing studies found age-associated microglial cells were characterized by activation of genes implicated in phagocytic activity and lipid metabolism in mice or genes involved in cell adhesion in humans (Galatro et al., 2017; Safaiyan et al., 2021). Those above changes of microglia during aging were demonstrated to play an important role in age-related declined myelin regeneration. More importantly, rejuvenating microglia in aged brains helps to myelin regeneration in injured brains in the aged mice (Ruckh et al., 2012; Cantuti-Castelvetri and Fitzner, 2018).

Astrocytes could secret many factors such as PDGF-A and FGF2 to facilitate OPCs proliferation and differentiation during myelin development (Rawji et al., 2020). In demyelinating diseases, astrocytes respond quickly by upregulating several proinflammatory cytokines, chemokines, as well as remyelination-signaling molecules (Williams et al., 2007). A recent study revealed that reactive astrocytes could be induced by inflammatory microglia cells and cause the death of neurons and OLs through producing saturated lipids (Liddelow et al., 2017; Guttenplan et al., 2021). In addition, astrocytes may also play a role in recruiting phagocytic microglia in areas of demyelination (Skripuletz et al., 2013). Aging astrocytes appear more reactive, displaying an upregulation in cytoskeletal proteins and hypertrophic cell bodies with shorter processes (Cerbai et al., 2012; Jyothi et al., 2015; Robillard et al., 2016). Consistent with morphological changes, RNA sequencing reveals that aging astrocytes also showed upregulation of reaction related genes, turning into an inflammatory state. Moreover, aging astrocytes were found to have a decrease in transcripts encoding cholesterol synthesis enzymes, which may induce a deficiency for myelin synthesis substrate (Boisvert et al., 2018; Clarke et al., 2018). These changes may be detrimental to OPC differentiation and myelin formation in the aged brain. Besides, other factors, including oxidative stress induced by vascular changes during aging and deregulation of glutamate neurotransmission in the aged brains may also play roles in age-related myelinogenesis decline (Rivera et al., 2016; Bagi et al., 2018; Bors et al., 2018; Sams, 2021; Summarized in Figure 1).

#### Perspectives

Age-related neurofunction decline may negatively impact the daily life for the elderly, and no effective strategies are available so far in clinic. As mentioned above, this present review mainly focuses on myelin degeneration, decreased myelinogenesis during aging and the possible mechanisms. Admittedly, there are a lot of questions remain unanswered. For instance, whether there is spatial- or temporal-differences in the degeneration process in the CNS? What is the deciding point for one OL or one myelin segment to initiate degenerate and could we inhibit this bad process through modulating one key factor? Whether the newly generated myelin is more stable compared to pre-existed myelin in aged brain and if this is the case, we may find some clues about repressing myelin degeneration in the aged. The decreased myelinogenesis during aging is likely a result of arrested OPCs differentiation, thus it is plausible that promoting adult OPCs maturation may be a feasible and realistic approach to improve age-related neuronal function decline for the elderly. Meanwhile, rejuvenating the SVZ stem cells may also help with myelinogenesis ability in the aged. More efforts are needed to further confirm those effects in human.

Moreover, oligodendroglial lineage cells display more than differentiation and forming new myelin sheaths. For example, OPCs may form synaptic connection with neighboring neurons, and that regulates neuronal signal in CNS. In addition, the expression of connexin channel proteins in oligodendroglial lineage cells is an intriguing feature and the connexins could function either as hemichannels or gap junctions. The gap junction enables OLs to be connected as a glial network with astrocytes, allowing transporting small molecules such as calcium and energy metabolites, which may be important for the homeostasis of the CNS. Recent studies even showed that OPCs could exert immunomodulatory functions, which are particularly relevant in the context of neurodegeneration and demyelinating diseases. Besides, OLs are found to be heterogenetic in the mouse juvenile and adult CNS (Marques et al., 2016; Chamling et al., 2021), the response of different subtypes to aging remains unknown. It is not clear whether the functions mentioned above and their correspondent molecules are altered during aging. Future works are needed to give us a more comprehensive understanding of the role oligodendroglial lineage cells played in aged brains, which could shed light on the clinical therapeutic strategies considering age-related neuronal functional diseases.

## Author Contributions

XZ prepared the manuscript. NH prepared the figure and revised the manuscript for grammatic typos. LX provided views and revised the manuscript. FW and TL designed the framework of the manuscript and prepared and finalized the manuscript. All authors agreed to approved the final manuscript.

## Conflict of Interest

The authors declare that the research was conducted in the absence of any commercial or financial relationships that could be construed as a potential conflict of interest.

## Publisher’s Note

All claims expressed in this article are solely those of the authors and do not necessarily represent those of their affiliated organizations, or those of the publisher, the editors and the reviewers. Any product that may be evaluated in this article, or claim that may be made by its manufacturer, is not guaranteed or endorsed by the publisher.

## References

[B1] Attia H. Taha M. Abdellatif A. (2019). Effects of aging on the myelination of the optic nerve in rats. *Int. J. Neurosci.* 129 320–324. 10.1080/00207454.2018.1529670 30260726

[B2] Bagi Z. Brandner D. D. Le P. McNeal D. W. Gong X. Dou H. (2018). Vasodilator dysfunction and oligodendrocyte dysmaturation in aging white matter. *Ann. Neurol.* 83 142–152. 10.1002/ana.25129 29283444 PMC5876126

[B3] Barrick T. R. Charlton R. A. Clark C. A. Markus H. S. (2010). White matter structural decline in normal ageing: a prospective longitudinal study using tract-based spatial statistics. *Neuroimage* 51 565–577. 10.1016/j.neuroimage.2010.02.033 20178850

[B4] Bartrés-Faz D. Clemente I. C. Junqué C. (2001). [White matter changes and cognitive performance in aging]. *Rev. Neurol.* 33 347–353. 11588730

[B5] Baumann N. Pham-Dinh D. (2001). Biology of oligodendrocyte and myelin in the mammalian central nervous system. *Physiol. Rev.* 81 871–927. 10.1152/physrev.2001.81.2.871 11274346

[B6] Baxi E. G. DeBruin J. Jin J. Strasburger H. J. Smith M. D. Orthmann-Murphy J. L. (2017). Lineage tracing reveals dynamic changes in oligodendrocyte precursor cells following cuprizone-induced demyelination. *Glia* 65 2087–2098. 10.1002/glia.23229 28940645 PMC5761347

[B7] Bells S. Lefebvre J. Longoni G. Narayanan S. Arnold D. L. Yeh E. A. (2019). White matter plasticity and maturation in human cognition. *Glia* 67 2020–2037. 10.1002/glia.23661 31233643

[B8] Bennett I. J. Madden D. J. (2014). Disconnected aging: cerebral white matter integrity and age-related differences in cognition. *Neuroscience* 276 187–205. 10.1016/j.neuroscience.2013.11.026 24280637 PMC4032380

[B9] Berghoff S. A. Spieth L. Sun T. Hosang L. Schlaphoff L. Depp C. (2021). Microglia facilitate repair of demyelinated lesions via post-squalene sterol synthesis. *Nat. Neurosci.* 24 47–60. 10.1038/s41593-020-00757-6 33349711 PMC7116742

[B10] Boisvert M. M. Erikson G. A. Shokhirev M. N. Allen N. J. (2018). The Aging Astrocyte Transcriptome from Multiple Regions of the Mouse Brain. *Cell Rep.* 22 269–285. 10.1016/j.celrep.2017.12.039 29298427 PMC5783200

[B11] Bors L. Tóth K. Tóth E. Z. Bajza Á Csorba A. Szigeti K. (2018). Age-dependent changes at the blood-brain barrier. A Comparative structural and functional study in young adult and middle aged rats. *Brain Res. Bull.* 139 269–277. 10.1016/j.brainresbull.2018.03.001 29522862

[B12] Bottes S. Jessberger S. (2021). Live imaging of remyelination in the adult mouse corpus callosum. *Proc. Natl. Acad. Sci. U S A.* 118:2025795118. 10.1073/pnas.2025795118 34244440 PMC8285919

[B13] Bouab M. Paliouras G. N. Aumont A. Forest-Bérard K. Fernandes K. J. (2011). Aging of the subventricular zone neural stem cell niche: evidence for quiescence-associated changes between early and mid-adulthood. *Neuroscience* 173 135–149. 10.1016/j.neuroscience.2010.11.032 21094223

[B14] Bowley M. P. Cabral H. Rosene D. L. Peters A. (2010). Age changes in myelinated nerve fibers of the cingulate bundle and corpus callosum in the rhesus monkey. *J. Comp. Neurol.* 518 3046–3064. 10.1002/cne.22379 20533359 PMC2889619

[B15] Caligiuri M. E. Perrotta P. Augimeri A. Rocca F. Quattrone A. Cherubini A. (2015). Automatic Detection of White Matter Hyperintensities in Healthy Aging and Pathology Using Magnetic Resonance Imaging: A Review. *Neuroinformatics* 13 261–276. 10.1007/s12021-015-9260-y 25649877 PMC4468799

[B16] Cantuti-Castelvetri L. Fitzner D. (2018). Defective cholesterol clearance limits remyelination in the aged central nervous system. *Science* 359 684–688. 10.1126/science.aan4183 29301957

[B17] Cerbai F. Lana D. Nosi D. Petkova-Kirova P. Zecchi S. Brothers H. M. (2012). The neuron-astrocyte-microglia triad in normal brain ageing and in a model of neuroinflammation in the rat hippocampus. *PLoS One* 7:e45250. 10.1371/journal.pone.0045250 23028880 PMC3445467

[B18] Chamling X. Kallman A. Fang W. Berlinicke C. A. Mertz J. L. Devkota P. (2021). Single-cell transcriptomic reveals molecular diversity and developmental heterogeneity of human stem cell-derived oligodendrocyte lineage cells. *Nat. Commun.* 12:652. 10.1038/s41467-021-20892-3 33510160 PMC7844020

[B19] Chang A. Nishiyama A. Peterson J. Prineas J. Trapp B. D. (2000). NG2-positive oligodendrocyte progenitor cells in adult human brain and multiple sclerosis lesions. *J. Neurosci.* 20 6404–6412. 10.1523/jneurosci.20-17-06404.2000 10964946 PMC6772992

[B20] Chapman T. W. Hill R. A. (2020). Myelin plasticity in adulthood and aging. *Neurosci. Lett.* 715:134645. 10.1016/j.neulet.2019.134645 31765728 PMC6981290

[B21] Chen L. Ren S. Y. Li R. X. Liu K. Chen J. F. Yang Y. J. (2021). Chronic Exposure to Hypoxia Inhibits Myelinogenesis and Causes Motor Coordination Deficits in Adult Mice. *Neurosci. Bull.* 2021 745–741. 10.1007/s12264-021-00745-1 34292513 PMC8490606

[B22] Chen J. F. Liu K. Hu B. Li R. R. Xin W. Chen H. (2021). Enhancing myelin renewal reverses cognitive dysfunction in a murine model of Alzheimer’s disease. *Neuron* 109 2292.e–2307.e. 10.1016/j.neuron.2021.05.012 34102111 PMC8298291

[B23] Cignarella F. Filipello F. Bollman B. Cantoni C. Locca A. Mikesell R. (2020). TREM2 activation on microglia promotes myelin debris clearance and remyelination in a model of multiple sclerosis. *Acta Neuropathol.* 140 513–534. 10.1007/s00401-020-02193-z 32772264 PMC7498497

[B24] Clarke L. E. Liddelow S. A. Chakraborty C. Münch A. E. Heiman M. Barres B. A. (2018). Normal aging induces A1-like astrocyte reactivity. *Proc. Natl. Acad. Sci. U S A.* 115 E1896–E1905. 10.1073/pnas.1800165115 29437957 PMC5828643

[B25] Coelho A. Fernandes H. M. Magalhães R. Moreira P. S. Marques P. Soares J. M. (2021). Signatures of white-matter microstructure degradation during aging and its association with cognitive status. *Sci. Rep.* 11:4517. 10.1038/s41598-021-83983-7 33633204 PMC7907273

[B26] Conway G. D. O’Bara M. A. Vedia B. H. Pol S. U. Sim F. J. (2012). Histone deacetylase activity is required for human oligodendrocyte progenitor differentiation. *Glia* 60 1944–1953. 10.1002/glia.22410 22927334

[B27] Cox S. R. Ritchie S. J. Tucker-Drob E. M. Liewald D. C. (2016). Ageing and brain white matter structure in 3,513 UK Biobank participants. *Nat. Commun.* 7:13629. 10.1038/ncomms13629 27976682 PMC5172385

[B28] Damoiseaux J. S. (2017). Effects of aging on functional and structural brain connectivity. *Neuroimage* 160 32–40. 10.1016/j.neuroimage.2017.01.077 28159687

[B29] de la Fuente A. Queiroz R. Ghosh T. McMurran C. Cubillos J. Bergles D. (2020). Changes in the Oligodendrocyte Progenitor Cell Proteome with Ageing. *Mol. Cell Proteomics* 19 1281–1302. 10.1074/mcp.RA120.002102 32434922 PMC8015006

[B30] Depp C. Sun T. Sasmita A. O. Spieth L. Berghoff S. A. Steixner-Kumar A. A. (2021). Ageing-associated myelin dysfunction drives amyloid deposition in mouse models of Alzheimer’s disease. [Preprint]. 10.1038/s41586-023-06120-6 PMC10247380 37258678

[B31] Deshmukh V. A. Tardif V. Lyssiotis C. A. Green C. C. Kerman B. Kim H. J. (2013). A regenerative approach to the treatment of multiple sclerosis. *Nature* 502 327–332. 10.1038/nature12647 24107995 PMC4431622

[B32] Ding S. Guo Y. Chen X. Du S. Han Y. Yan Z. (2021). Demyelination and remyelination detected in an alternative cuprizone mouse model of multiple sclerosis with 7.0 T multiparameter magnetic resonance imaging. *Sci. Rep.* 11:11060. 10.1038/s41598-021-90597-6 34040141 PMC8155133

[B33] Egawa N. Shindo A. Hikawa R. Kinoshita H. Liang A. C. Itoh K. (2019). Differential roles of epigenetic regulators in the survival and differentiation of oligodendrocyte precursor cells. *Glia* 67 718–728. 10.1002/glia.23567 30793389 PMC6573028

[B34] Elbaz B. Popko B. (2019). Molecular Control of Oligodendrocyte Development. *Trends Neurosci.* 42 263–277. 10.1016/j.tins.2019.01.002 30770136 PMC7397568

[B35] Emery B. (2010). Regulation of oligodendrocyte differentiation and myelination. *Science* 330 779–782. 10.1126/science.1190927 21051629

[B36] Emery B. Agalliu D. Cahoy J. D. Watkins T. A. Dugas J. C. Mulinyawe S. B. (2009). Myelin gene regulatory factor is a critical transcriptional regulator required for CNS myelination. *Cell* 138 172–185. 10.1016/j.cell.2009.04.031 19596243 PMC2757090

[B37] Franklin R. Ffrench-Constant C. J. N. R. N. (2008). Remyelination in the CNS: from biology to therapy. *Nat. Rev. Neurosci.* 9 839–855. 10.1038/nrn2480 18931697

[B38] Fünfschilling U. Supplie L. M. Mahad D. Boretius S. Saab A. S. Edgar J. (2012). Glycolytic oligodendrocytes maintain myelin and long-term axonal integrity. *Nature* 485 517–521. 10.1038/nature11007 22622581 PMC3613737

[B39] Galatro T. F. Holtman I. R. Lerario A. M. Vainchtein I. D. Brouwer N. Sola P. R. (2017). Transcriptomic analysis of purified human cortical microglia reveals age-associated changes. *Nat. Neurosci.* 20 1162–1171. 10.1038/nn.4597 28671693

[B40] Gazzaley A. Cooney J. W. Rissman J. D’Esposito M. (2005). Top-down suppression deficit underlies working memory impairment in normal aging. *Nat. Neurosci.* 8 1298–1300. 10.1038/nn1543 16158065

[B41] Gibson E. M. Purger D. Mount C. W. Goldstein A. K. Lin G. L. Wood L. S. (2014). Neuronal activity promotes oligodendrogenesis and adaptive myelination in the mammalian brain. *Science* 344:1252304. 10.1126/science.1252304 24727982 PMC4096908

[B42] Giera S. Luo R. Ying Y. Ackerman S. D. Jeong S. J. Stoveken H. M. (2018). Microglial transglutaminase-2 drives myelination and myelin repair via GPR56/ADGRG1 in oligodendrocyte precursor cells. *Elife* 7:33385. 10.7554/eLife.33385 29809138 PMC5980231

[B43] Gilson J. Blakemore W. F. (1993). Failure of remyelination in areas of demyelination produced in the spinal cord of old rats. *Neuropathol. Appl. Neurobiol.* 19 173–181. 10.1111/j.1365-2990.1993.tb00424.x 8316337

[B44] Goldschmidt T. Antel J. Konig F. B. Bruck W. Kuhlmann T. (2009). Remyelination capacity of the MS brain decreases with disease chronicity. *Neurology* 72 1914–1921. 10.1212/WNL.0b013e3181a8260a 19487649

[B45] Guttenplan K. A. Weigel M. K. Prakash P. Wijewardhane P. R. Hasel P. Rufen-Blanchette U. (2021). Neurotoxic reactive astrocytes induce cell death via saturated lipids. *Nature* 2021:03960–y. 10.1038/s41586-021-03960-y 34616039 PMC12054010

[B46] Hase Y. Horsburgh K. Ihara M. Kalaria R. N. (2018). White matter degeneration in vascular and other ageing-related dementias. *J. Neurochem.* 144 617–633. 10.1111/jnc.14271 29210074

[B47] Hill R. A. Li A. M. Grutzendler J. (2018). Lifelong cortical myelin plasticity and age-related degeneration in the live mammalian brain. *Nat. Neurosci.* 21 683–695. 10.1038/s41593-018-0120-6 29556031 PMC5920745

[B48] Hinman J. D. Chen C. D. Oh S. Y. Hollander W. Abraham C. R. (2008). Age-dependent accumulation of ubiquitinated 2’,3’-cyclic nucleotide 3’-phosphodiesterase in myelin lipid rafts. *Glia* 56 118–133. 10.1002/glia.20595 17963267

[B49] Hinman J. D. Peters A. Cabral H. Rosene D. L. Hollander W. Rasband M. N. (2006). Age-related molecular reorganization at the node of Ranvier. *J. Comp. Neurol.* 495 351–362. 10.1002/cne.20886 16485288 PMC4444368

[B50] Hughes A. N. Appel B. (2020). Microglia phagocytose myelin sheaths to modify developmental myelination. *Nat. Neurosci.* 23 1055–1066. 10.1038/s41593-020-0654-2 32632287 PMC7483351

[B51] Hughes E. G. Orthmann-Murphy J. L. (2018). Myelin remodeling through experience-dependent oligodendrogenesis in the adult somatosensory cortex. *Nat. Neurosci.* 21 696–706. 10.1038/s41593-018-0121-5 29556025 PMC5920726

[B52] Hughes E. G. Kang S. H. Fukaya M. Bergles D. E. (2013). Oligodendrocyte progenitors balance growth with self-repulsion to achieve homeostasis in the adult brain. *Nat. Neurosci.* 16 668–676. 10.1038/nn.3390 23624515 PMC3807738

[B53] Ihara R. Vincent B. D. Baxter M. R. Franklin E. E. Hassenstab J. J. Xiong C. (2018). Relative neuron loss in hippocampal sclerosis of aging and Alzheimer’s disease. *Ann. Neurol.* 84 741–753. 10.1002/ana.25344 30246887 PMC6373729

[B54] Ito M. Muramatsu R. Kato Y. Sharma B. Uyeda A. Tanabe S. (2021). Age-dependent decline in remyelination capacity is mediated by apelin–APJ signaling. *Nat. Aging* 1 284–294. 10.1038/s43587-021-00041-7 37118408

[B55] Jeromin A. Bowser R. (2017). Biomarkers in Neurodegenerative Diseases. *Adv. Neurobiol.* 15 491–528. 10.1007/978-3-319-57193-5_20 28674995

[B56] Jin J. Peng Q. Hou Z. Jiang M. Wang X. Langseth A. J. (2015). Early white matter abnormalities, progressive brain pathology and motor deficits in a novel knock-in mouse model of Huntington’s disease. *Hum. Mol. Genet.* 24 2508–2527. 10.1093/hmg/ddv016 25609071 PMC4383863

[B57] Jyothi H. J. Vidyadhara D. J. Mahadevan A. Philip M. Parmar S. K. Manohari S. G. (2015). Aging causes morphological alterations in astrocytes and microglia in human substantia nigra pars compacta. *Neurobiol. Aging* 36 3321–3333. 10.1016/j.neurobiolaging.2015.08.024 26433682

[B58] Keough M. B. Rogers J. A. Zhang P. Jensen S. K. Stephenson E. L. Chen T. (2016). An inhibitor of chondroitin sulfate proteoglycan synthesis promotes central nervous system remyelination. *Nat. Commun.* 7:11312. 10.1038/ncomms11312 27115988 PMC4853428

[B59] Kohama S. G. Rosene D. L. Sherman L. S. (2012). Age-related changes in human and non-human primate white matter: from myelination disturbances to cognitive decline. *Age* 34 1093–1110. 10.1007/s11357-011-9357-7 22203458 PMC3448998

[B60] Kohnke S. Buller S. Nuzzaci D. Ridley K. Lam B. Pivonkova H. (2021). Nutritional regulation of oligodendrocyte differentiation regulates perineuronal net remodeling in the median eminence. *Cell Rep.* 36:109362. 10.1016/j.celrep.2021.109362 34260928 PMC8293628

[B61] Kolb H. Absinta M. Beck E. S. Ha S. K. Song Y. Norato G. (2021). 7T MRI Differentiates Remyelinated from Demyelinated Multiple Sclerosis Lesions. *Ann. Neurol.* 90 612–626. 10.1002/ana.26194 34390015 PMC9291186

[B62] Lasiene J. Matsui A. Sawa Y. Wong F. Horner P. J. (2009). Age-related myelin dynamics revealed by increased oligodendrogenesis and short internodes. *Aging Cell* 8 201–213. 10.1111/j.1474-9726.2009.00462.x 19338498 PMC2703583

[B63] Lee Y. Morrison B. M. Li Y. Lengacher S. Farah M. H. Hoffman P. N. (2012). Oligodendroglia metabolically support axons and contribute to neurodegeneration. *Nature* 487 443–448. 10.1038/nature11314 22801498 PMC3408792

[B64] Liddelow S. A. Guttenplan K. A. Clarke L. E. Bennett F. C. Bohlen C. J. Schirmer L. (2017). Neurotoxic reactive astrocytes are induced by activated microglia. *Nature* 541 481–487. 10.1038/nature21029 28099414 PMC5404890

[B65] Liu H. Hu Q. D’Ercole A. J. Ye P. (2009). Histone deacetylase 11 regulates oligodendrocyte-specific gene expression and cell development in OL-1 oligodendroglia cells. *Glia* 57 1–12. 10.1002/glia.20729 18627006 PMC2595137

[B66] Liu H. Wang L. Geng Z. Zhu Q. Song Z. Chang R. (2016). A voxel-based morphometric study of age- and sex-related changes in white matter volume in the normal aging brain. *Neuropsychiatr. Dis. Treat.* 12 453–465. 10.2147/ndt.S90674 26966366 PMC4771405

[B67] Liu H. Yang Y. Xia Y. Zhu W. Leak R. K. Wei Z. (2017). Aging of cerebral white matter. *Ageing Res. Rev.* 34 64–76. 10.1016/j.arr.2016.11.006 27865980 PMC5250573

[B68] Luo J. Daniels S. B. Lennington J. B. Notti R. Q. Conover J. C. (2006). The aging neurogenic subventricular zone. *Aging Cell* 5 139–152. 10.1111/j.1474-9726.2006.00197.x 16626393

[B69] Macke E. L. Henningsen E. Jessen E. Zumwalde N. A. Landowski M. Western D. E. (2020). Loss of Chondroitin Sulfate Modification Causes Inflammation and Neurodegeneration in skt Mice. *Genetics* 214 121–134. 10.1534/genetics.119.302834 31754016 PMC6944401

[B70] Marques S. Zeisel A. Codeluppi S. van Bruggen D. Mendanha Falcão A. Xiao L. (2016). Oligodendrocyte heterogeneity in the mouse juvenile and adult central nervous system. *Science* 352 1326–1329. 10.1126/science.aaf6463 27284195 PMC5221728

[B71] Marschallinger J. Iram T. Zardeneta M. Lee S. E. Lehallier B. Haney M. S. (2019). Lipid droplet accumulating microglia represent a dysfunctional and pro-inflammatory state in the aging brain. *Nat. Neurosci.* 23 194–208. 10.1038/s41593-019-0566-1 PMC7595134 31959936

[B72] McKenzie I. A. Ohayon D. Li H. de Faria J. P. Emery B. Tohyama K. (2014). Motor skill learning requires active central myelination. *Science* 346 318–322. 10.1126/science.1254960 25324381 PMC6324726

[B73] Mei F. Lehmann-Horn K. Shen Y. A. Rankin K. A. Stebbins K. J. Lorrain D. S. (2016). Accelerated remyelination during inflammatory demyelination prevents axonal loss and improves functional recovery. *Elife* 5 1–21. 10.7554/eLife.18246 27671734 PMC5039026

[B74] Mei F. Wang H. Liu S. Niu J. Wang L. He Y. (2013). Stage-specific deletion of Olig2 conveys opposing functions on differentiation and maturation of oligodendrocytes. *J. Neurosci.* 33 8454–8462. 10.1523/jneurosci.2453-12.2013 23658182 PMC3865513

[B75] Menn B. Garcia-Verdugo J. M. Yaschine C. Gonzalez-Perez O. Rowitch D. Alvarez-Buylla A. (2006). Origin of oligodendrocytes in the subventricular zone of the adult brain. *J. Neurosci.* 26 7907–7918. 10.1523/jneurosci.1299-06.2006 16870736 PMC6674207

[B76] Morrison B. M. Lee Y. Rothstein J. D. (2013). Oligodendroglia: metabolic supporters of axons. *Trends Cell Biol.* 23 644–651. 10.1016/j.tcb.2013.07.007 23988427 PMC3842360

[B77] Morrison J. H. Hof P. R. (1997). Life and death of neurons in the aging brain. *Science* 278 412–419. 10.1126/science.278.5337.412 9334292

[B78] Mukoyama M. (1973). Age changes in internodal length in the human spinal roots–nerve teasing study. *Nagoya J. Med. Sci.* 36 17–27. 4592300

[B79] Nasrabady S. E. Rizvi B. Goldman J. E. Brickman A. M. (2018). White matter changes in Alzheimer’s disease: a focus on myelin and oligodendrocytes. *Acta Neuropathol. Commun.* 6:22. 10.1186/s40478-018-0515-3 29499767 PMC5834839

[B80] Neely S. A. Williamson J. M. Klingseisen A. Zoupi L. Early J. J. Williams A. (2020). New oligodendrocytes exhibit more abundant and accurate myelin regeneration than those that survive demyelination. [Preprint]. 10.1038/s41593-021-01009-x PMC7612594 35165460

[B81] Neumann B. Baror R. Zhao C. Segel M. Dietmann S. Rawji K. S. (2019a). Metformin Restores CNS Remyelination Capacity by Rejuvenating Aged Stem Cells. *Cell Stem Cell* 25 473.e–485.e. 10.1016/j.stem.2019.08.015 31585093 PMC6863391

[B82] Neumann B. Segel M. Chalut K. J. Franklin R. J. (2019b). Remyelination and ageing: Reversing the ravages of time. *Mult. Scler.* 25 1835–1841. 10.1177/1352458519884006 31687878 PMC7682531

[B83] Pakkenberg B. Gundersen H. J. (1997). Neocortical neuron number in humans: effect of sex and age. *J. Comp. Neurol.* 384 312–320. 10.1002/(sici)1096-9861(19970728)384:2<312::aid-cne10>3.0.co;2-k 9215725

[B84] Pan S. Mayoral S. R. Choi H. S. Chan J. R. Ma K. (2020). Preservation of a remote fear memory requires new myelin formation. *Neuron* 23 487–499. 10.1038/s41593-019-0582-1 32042175 PMC7213814

[B85] Philips T. Mironova Y. A. Jouroukhin Y. Chew J. Vidensky S. Farah M. H. (2021). MCT1 Deletion in Oligodendrocyte Lineage Cells Causes Late-Onset Hypomyelination and Axonal Degeneration. *Cell Rep.* 34:108610. 10.1016/j.celrep.2020.108610 33440165 PMC8020895

[B86] Phillips K. A. Watson C. M. Bearman A. Knippenberg A. R. Adams J. Ross C. (2019). Age-related changes in myelin of axons of the corpus callosum and cognitive decline in common marmosets. *Am. J. Primatol.* 81:e22949. 10.1002/ajp.22949 30620098 PMC6685070

[B87] Pini L. Pievani M. Bocchetta M. Altomare D. Bosco P. Cavedo E. (2016). Brain atrophy in Alzheimer’s Disease and aging. *Ageing Res. Rev.* 30 25–48. 10.1016/j.arr.2016.01.002 26827786

[B88] Poliani P. L. Wang Y. Fontana E. Robinette M. L. Yamanishi Y. Gilfillan S. (2015). TREM2 sustains microglial expansion during aging and response to demyelination. *J. Clin. Invest.* 125 2161–2170. 10.1172/jci77983 25893602 PMC4463196

[B89] Purger D. Gibson E. M. Monje M. (2016). Myelin plasticity in the central nervous system. *Neuropharmacology* 110 (Pt B), 563–573. 10.1016/j.neuropharm.2015.08.001 26282119

[B90] Rawji K. S. Gonzalez Martinez G. A. Sharma A. Franklin R. J. M. (2020). The Role of Astrocytes in Remyelination. *Trends Neurosci.* 43 596–607. 10.1016/j.tins.2020.05.006 32620289

[B91] Rawji K. S. Kappen J. Tang W. Teo W. Plemel J. R. Stys P. K. (2018). Deficient Surveillance and Phagocytic Activity of Myeloid Cells Within Demyelinated Lesions in Aging Mice Visualized by Ex Vivo Live Multiphoton Imaging. *J. Neurosci.* 38 1973–1988. 10.1523/jneurosci.2341-17.2018 29363580 PMC6705888

[B92] Reich D. S. Lucchinetti C. F. Calabresi P. A. (2018). Multiple Sclerosis. *N. Engl. J. Med.* 378 169–180. 10.1056/NEJMra1401483 29320652 PMC6942519

[B93] Richard A. D. Tian X. L. El-Saadi M. W. Lu X. H. (2018). Erasure of striatal chondroitin sulfate proteoglycan-associated extracellular matrix rescues aging-dependent decline of motor learning. *Neurobiol. Aging* 71 61–71. 10.1016/j.neurobiolaging.2018.07.008 30099347

[B94] Rivera A. D. Pieropan F. Chacon-De-La-Rocha I. Lecca D. Abbracchio M. P. Azim K. (2021). Functional genomic analyses highlight a shift in Gpr17-regulated cellular processes in oligodendrocyte progenitor cells and underlying myelin dysregulation in the aged mouse cerebrum. *Aging Cell* 20:e13335. 10.1111/acel.13335 33675110 PMC8045941

[B95] Rivera A. Vanzuli I. Arellano J. J. Butt A. (2016). Decreased Regenerative Capacity of Oligodendrocyte Progenitor Cells (NG2-Glia) in the Ageing Brain: A Vicious Cycle of Synaptic Dysfunction, Myelin Loss and Neuronal Disruption? *Curr. Alzheimer. Res.* 13 413–418. 10.2174/1567205013666151116125518 26567743

[B96] Robillard K. N. Lee K. M. Chiu K. B. MacLean A. G. (2016). Glial cell morphological and density changes through the lifespan of rhesus macaques. *Brain Behav. Immun.* 55 60–69. 10.1016/j.bbi.2016.01.006 26851132 PMC4899176

[B97] Ruckh J. M. Zhao J. W. Shadrach J. L. van Wijngaarden P. Rao T. N. Wagers A. J. (2012). Rejuvenation of regeneration in the aging central nervous system. *Cell Stem Cell* 10 96–103. 10.1016/j.stem.2011.11.019 22226359 PMC3714794

[B98] Safaiyan S. Besson-Girard S. Kaya T. Cantuti-Castelvetri L. Liu L. Ji H. (2021). White matter aging drives microglial diversity. *Neuron* 109 1100.e–1117.e. 10.1016/j.neuron.2021.01.027 33606969

[B99] Safaiyan S. Kannaiyan N. Snaidero N. Brioschi S. Biber K. Yona S. (2016). Age-related myelin degradation burdens the clearance function of microglia during aging. *Nat. Neurosci.* 19 995–998. 10.1038/nn.4325 27294511 PMC7116794

[B100] Sams E. C. (2021). Oligodendrocytes in the aging brain. *Neuronal Signal* 5:Ns20210008. 10.1042/ns20210008 34290887 PMC8264650

[B101] Segel M. Neumann B. Hill M. F. E. Weber I. P. Viscomi C. Zhao C. (2019). Niche stiffness underlies the ageing of central nervous system progenitor cells. *Nature* 573 130–134. 10.1038/s41586-019-1484-9 31413369 PMC7025879

[B102] Shen S. Li J. Casaccia-Bonnefil P. (2005). Histone modifications affect timing of oligodendrocyte progenitor differentiation in the developing rat brain. *J. Cell Biol.* 169 577–589. 10.1083/jcb.200412101 15897262 PMC2171688

[B103] Shen S. Sandoval J. Swiss V. A. Li J. Dupree J. Franklin R. J. (2008). Age-dependent epigenetic control of differentiation inhibitors is critical for remyelination efficiency. *Nat. Neurosci.* 11 1024–1034. 10.1038/nn.2172 19160500 PMC2656679

[B104] Shepherd M. N. Pomicter A. D. Velazco C. S. Henderson S. C. Dupree J. L. (2012). Paranodal reorganization results in the depletion of transverse bands in the aged central nervous system. *Neurobiol. Aging* 33 .e213–.e224. 10.1016/j.neurobiolaging.2010.08.001 20888080 PMC3282488

[B105] Sim F. J. Zhao C. Penderis J. Franklin R. J. (2002). The age-related decrease in CNS remyelination efficiency is attributable to an impairment of both oligodendrocyte progenitor recruitment and differentiation. *J. Neurosci.* 22 2451–2459. 10.1523/JNEUROSCI.22-07-02451.2002 11923409 PMC6758320

[B106] Skripuletz T. Hackstette D. Bauer K. Gudi V. Pul R. Voss E. (2013). Astrocytes regulate myelin clearance through recruitment of microglia during cuprizone-induced demyelination. *Brain* 136 (Pt 1), 147–167. 10.1093/brain/aws262 23266461

[B107] Sloane J. A. Hinman J. D. Lubonia M. Hollander W. Abraham C. R. (2003). Age-dependent myelin degeneration and proteolysis of oligodendrocyte proteins is associated with the activation of calpain-1 in the rhesus monkey. *J. Neurochem.* 84 157–168. 10.1046/j.1471-4159.2003.01541.x 12485412

[B108] Smith K. J. Blakemore W. F. McDonald W. I. (1981). The restoration of conduction by central remyelination. *Brain* 104 383–404. 10.1093/brain/104.2.383 6263406

[B109] Sousounis K. Baddour J. A. Tsonis P. A. (2014). Aging and regeneration in vertebrates. *Curr. Top. Dev. Biol.* 108 217–246. 10.1016/b978-0-12-391498-9.00008-5 24512711

[B110] Spitzer S. O. Sitnikov S. Kamen Y. Evans K. A. Kronenberg-Versteeg D. Dietmann S. (2019). Oligodendrocyte Progenitor Cells Become Regionally Diverse and Heterogeneous with Age. *Neuron* 101 459.e–471.e. 10.1016/j.neuron.2018.12.020 30654924 PMC6372724

[B111] Stadelmann C. Timmler S. Barrantes-Freer A. Simons M. (2019). Myelin in the Central Nervous System: Structure, Function, and Pathology. *Physiol. Rev.* 99 1381–1431. 10.1152/physrev.00031.2018 31066630

[B112] Steadman P. E. Xia F. Ahmed M. Mocle A. J. Penning A. R. A. Geraghty A. C. (2020). Disruption of Oligodendrogenesis Impairs Memory Consolidation in Adult Mice. *Neuron* 105 150.e–164.e. 10.1016/j.neuron.2019.10.013 31753579 PMC7579726

[B113] Stephenson E. L. Zhang P. Ghorbani S. Wang A. Gu J. Keough M. B. (2019). Targeting the Chondroitin Sulfate Proteoglycans: Evaluating Fluorinated Glucosamines and Xylosides in Screens Pertinent to Multiple Sclerosis. *ACS Cent. Sci.* 5 1223–1234. 10.1021/acscentsci.9b00327 31404231 PMC6661872

[B114] Streit W. J. Sammons N. W. Kuhns A. J. Sparks D. L. (2004). Dystrophic microglia in the aging human brain. *Glia* 45 208–212. 10.1002/glia.10319 14730714

[B115] Sugiyama I. Tanaka K. Akita M. Yoshida K. Kawase T. Asou H. (2002). Ultrastructural analysis of the paranodal junction of myelinated fibers in 31-month-old-rats. *J. Neurosci. Res.* 70 309–317. 10.1002/jnr.10386 12391590

[B116] Sun L. O. Mulinyawe S. B. Collins H. Y. Ibrahim A. Li Q. Simon D. J. (2018). Spatiotemporal Control of CNS Myelination by Oligodendrocyte Programmed Cell Death through the TFEB-PUMA Axis. *Cell* 175 1811.e–1826.e. 10.1016/j.cell.2018.10.044 30503207 PMC6295215

[B117] Tang Y. Nyengaard J. R. Pakkenberg B. Gundersen H. J. (1997). Age-induced white matter changes in the human brain: a stereological investigation. *Neurobiol. Aging* 18 609–615. 10.1016/s0197-4580(97)00155-3 9461058

[B118] Tripathi R. B. Jackiewicz M. McKenzie I. A. Kougioumtzidou E. Grist M. Richardson W. D. (2017). Remarkable Stability of Myelinating Oligodendrocytes in Mice. *Cell Rep.* 21 316–323. 10.1016/j.celrep.2017.09.050 29020619 PMC5643547

[B119] Tse K. H. Herrup K. (2017). DNA damage in the oligodendrocyte lineage and its role in brain aging. *Mech. Ageing Dev.* 161 (Pt A), 37–50. 10.1016/j.mad.2016.05.006 27235538 PMC5124419

[B120] von Bartheld C. S. Bahney J. Herculano-Houzel S. (2016). The search for true numbers of neurons and glial cells in the human brain: A review of 150 years of cell counting. *J. Comp. Neurol.* 524 3865–3895. 10.1002/cne.24040 27187682 PMC5063692

[B121] Wang F. Ren S.-Y. Chen J.-F. Liu K. Li R.-X. Li Z.-F. (2020). Myelin degeneration and diminished myelin renewal contribute to age-related deficits in memory. *Nat. Neurosci.* 23 481–486. 10.1038/s41593-020-0588-8 32042174 PMC7306053

[B122] Wang F. Yang Y. J. Yang N. Chen X. J. Huang N. X. Zhang J. (2018). Enhancing Oligodendrocyte Myelination Rescues Synaptic Loss and Improves Functional Recovery after Chronic Hypoxia. *Neuron* 99 689–701e685. 10.1016/j.neuron.2018.07.017 30078577 PMC6170028

[B123] Wang H. Lian K. Han B. Wang Y. Kuo S. H. Geng Y. (2014). Age-related alterations in the metabolic profile in the hippocampus of the senescence-accelerated mouse prone 8: a spontaneous Alzheimer’s disease mouse model. *J. Alzheimers Dis.* 39 841–848. 10.3233/jad-131463 24284365 PMC4532290

[B124] Wang S. S. Shultz J. R. Burish M. J. Harrison K. H. Hof P. R. Towns L. C. (2008). Functional trade-offs in white matter axonal scaling. *J. Neurosci.* 28 4047–4056. 10.1523/jneurosci.5559-05.2008 18400904 PMC2779774

[B125] Williams A. Piaton G. Lubetzki C. (2007). Astrocytes–friends or foes in multiple sclerosis? *Glia* 55 1300–1312. 10.1002/glia.20546 17626262

[B126] Xiao L. Ohayon D. McKenzie I. A. Sinclair-Wilson A. Wright J. L. Fudge A. D. (2016). Rapid production of new oligodendrocytes is required in the earliest stages of motor-skill learning. *Nat. Neurosci.* 19 1210–1217. 10.1038/nn.4351 27455109 PMC5008443

[B127] Yeung M. S. Zdunek S. Bergmann O. Bernard S. Salehpour M. Alkass K. (2014). Dynamics of oligodendrocyte generation and myelination in the human brain. *Cell* 159 766–774. 10.1016/j.cell.2014.10.011 25417154

[B128] Young K. M. Psachoulia K. Tripathi R. B. Dunn S. J. Cossell L. Attwell D. (2013). Oligodendrocyte dynamics in the healthy adult CNS: evidence for myelin remodeling. *Neuron* 77 873–885. 10.1016/j.neuron.2013.01.006 23473318 PMC3842597

[B129] Yu Y. Chen Y. Kim B. Wang H. Zhao C. He X. (2013). Olig2 targets chromatin remodelers to enhancers to initiate oligodendrocyte differentiation. *Cell* 152 248–261. 10.1016/j.cell.2012.12.006 23332759 PMC3553550

[B130] Zhou J. Wu Y. C. Xiao B. J. Guo X. D. Zheng Q. X. Wu B. (2019). Age-related Changes in the Global DNA Methylation Profile of Oligodendrocyte Progenitor Cells Derived from Rat Spinal Cords. *Curr. Med Sci.* 39 67–74. 10.1007/s11596-019-2001-y 30868493

[B131] Zilkha-Falb R. Kaushansky N. Ben-Nun A. (2020). The Median Eminence, A New Oligodendrogenic Niche in the Adult Mouse Brain. *Stem Cell Rep.* 14 1076–1092. 10.1016/j.stemcr.2020.04.005 32413277 PMC7355143

